# Highly switchable and reversible dry adhesion for transfer printing

**DOI:** 10.1093/nsr/nwz134

**Published:** 2019-09-12

**Authors:** Yonggang Huang

**Affiliations:** Departments of Civil and Environmental Engineering, Mechanical Engineering, and Materials Science and Engineering, Center for Bio-Integrated Electronics, Northwestern University, USA

As an emerging assembly technique to transfer micro/nano-objects (i.e. inks) from donor substrate to receiver substrate using soft polymeric stamps, transfer printing has become increasingly popular in the last decade due to its unique capability of integrating diverse materials in various structural layouts. A growing number of applications are enabled by transfer printing, particular advanced flexible and stretchable electronic systems requiring heterogeneous integration of inorganic materials on plastic or rubber substrates [1]. The critical challenge for efficient transfer printing is to manipulate the stamp/ink interfacial adhesion between strong state for picking and weak state for printing in a rapid, repeatable, and robust manner. The kinetic approach that exploits rate-dependent viscosity effect through peeling velocity is very useful, but the adhesion switchability is low with the minimum adhesion usually larger than desired. Several other strategies that can complement or be combined with that based on rate have been demonstrated such as surface relief-assisted transfer printing [2] and magnetic-controlled transfer printing [3]. The recently developed laser-driven transfer printing [4] based on interfacial thermal mismatch eliminates the influence of receiver and allows non-contact printing of inks onto arbitrary receivers. However, the induced undesired high temperature usually larger than 200^o^C causes interfacial damages and greatly limits its broad utility.

**Figure 1.**
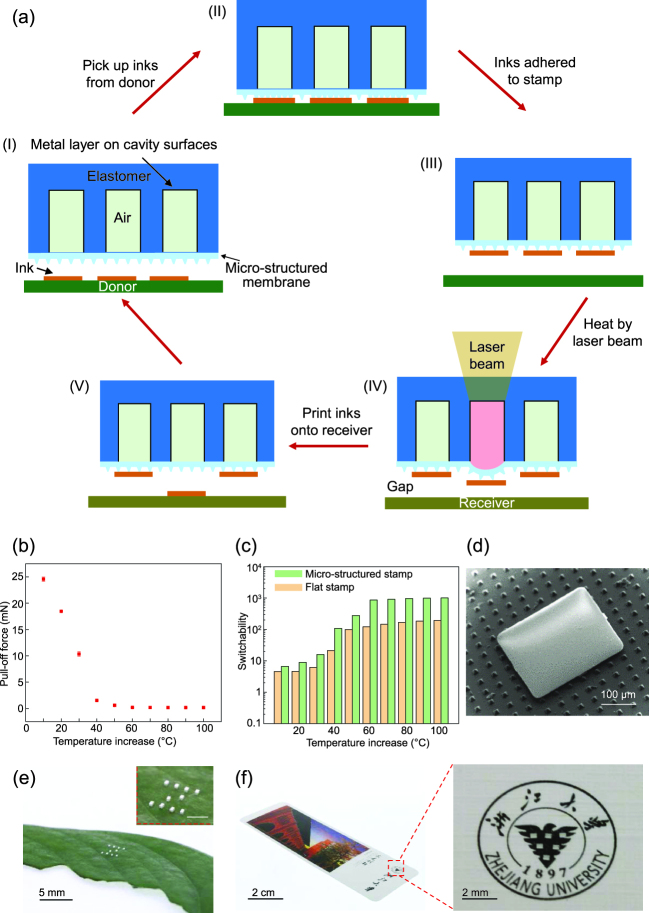
A laser-driven non-contact transfer printing technique based on an active elastomeric micro-structured stamp with tunable adhesion. (a) Schematic illustration of the transfer printing process. (b) The pull-off force as the function of the temperature increase. (c) The adhesion switchability versus the temperature increase. (d) Printing a single Si platelet onto PDMS with pyramid microstructures. (g) Programmable printing LED chips onto (e) a leaf to form a letter ‘z’ and (f) a postcard with a triangle pattern. Reprinted from Ref. [ 5] with permission of Oxford University Press.

In a recent work published in NSR by Luo *et al*. [5] ‘Laser-driven programm able non-contact transfer printing of objects onto arbitrary receivers via an active elastomeric micro-structured stamp’, a novel laser-driven programmable non-contact transfer printing (Fig. 1a) is developed based on a simple yet robust design of active elastomeric micro-structured stamp with tunable adhesion. The proposed stamp features three delicate components including the air-filled cavities, the micro-patterned encapsulation membrane, and the laser absorbing metal layer on the inner cavity (Fig. 1a–I). The heating of air in cavities through the laser absorbing metal layer inflates the micro-patterned membrane dynamically to modulate the interfacial adhesion (Fig. 1b). This construct yields a thermally controlled tunable adhesion with a large switchability of more than 10^3^ with a temperature increase below 100^o^C (Fig. 1c). The low temperature increase does not cause any surface degradation and ensures a highly reversible level of adhesion. The active dry adhesive developed by Luo *et al.* [5] outperforms most of existing ones and enables the development of a novel laser-driven programmable non-contact transfer printing technique. Demonstrations of programmable transfer printing of micro-scale Si platelets and micro-scale light-emitting diode (LED) chips onto various challenging receivers, such as polydimethylsiloxane (PDMS) with pyramid microstructures (Fig. 1d), a leaf (Fig. 1e), and a postcard (Fig. 1f), illustrate unusual capabilities for deterministic assembly.

In short, Luo *et al.* [5] proposed an innovative design of active dry adhesive for developing a laser-driven programmable non-contact transfer printing technique. The active dry adhesive offers a large strong/weak adhesion switchability in a rapid, repeatable, and robust manner, which meets the requirement of efficient transfer printing. This highly inspiring work partly narrows the gap between traditional semiconductor fabrication technologies and future engineering fabrications for advanced electronic systems such as flexible electronics, paper-based electronics, bio-integrated electronics, and microLED display, where the hete rogeneous integration of diverse materials is required.
